# Leukocytosis and presence of *CALR* mutation is associated with non-hepatosplenic extramedullary hematopoiesis in primary myelofibrosis

**DOI:** 10.1038/bcj.2016.44

**Published:** 2016-06-17

**Authors:** D Barraco, T L Lasho, N Gangat, C Finke, Y C Elala, A Pardanani, A Tefferi

**Affiliations:** 1 Division of Hematology, Department of Medicine, Mayo Clinic, Rochester, MN, USA

Extramedullary hematopoiesis (EMH) is a cardinal feature of primary myelofibrosis (PMF). The sites most frequently involved by EMH in PMF are the liver and spleen, leading to organ enlargement; however, many other organs have also been affected by EMH.^1^ Non-hepatosplenic EMH (NHS-EMH) has been reported in a multitude of tissues and organs, presenting as either an incidental finding or with symptomatic consequences. Pathogenetic mechanisms for PMF-associated EMH might include abnormal trafficking of clonal hematopoietic progenitor/stem cells further compounded by aberrant inflammatory cytokine production and abnormal host immune reaction.^2^ The current study was designed to evaluate and identify clinical and molecular markers of NHS-EMH in PMF in order to gain better insight into its pathogenesis and facilitate the recognition of patients that could develop NHS-EMH.

The current study was approved by the institutional review board of the Mayo Clinic. Clinical and laboratory data were collected from patients at the time of diagnosis or referral to the Mayo Clinic. The diagnosis of PMF was according to the World Health Organization criteria^3^ and risk stratification was assessed according to dynamic international prognostic scoring system-plus score.^4^ Screening for *JAK2*, *CALR*, *MPL*, *ASXL1*, *TET2*, *EZH2*, *IDH1*, *IDH2* and spliceosome (*SF3B1*, *U2AF1*, *SRSF2* and *ZRSR2*) mutations was performed according to previously described methods.^5, 6, 7^

The diagnosis of NHS-EMH was performed with radionuclide bone marrow scanning, tissue biopsy or fine-needle aspiration biopsy. Diagnosis of NHS-EMH performed with a computerized tomography or magnetic resonance imaging but without biopsy or radionuclide scan were excluded. Statistical analyses considered clinical and laboratory data collected at the time of diagnosis or referral to the Mayo Clinic. Differences in the distribution of continuous variables between categories were analyzed by either Mann–Whitney (for comparison of two groups) or Kruskal–Wallis (comparison of three or more groups) test. Patient groups with nominal variables were compared by *χ*^2^-test. Cox proportional hazard regression model was used for multivariable analysis. *P*-values <0.05 were considered significant. The Stat View (SAS Institute, Cary, NC, USA) statistical package was used for all calculations.

A total of 704 patients with PMF were studied, and their clinical and laboratory characteristics are listed in Table 1. The median age was 64 years and 64% were males. Dynamic international prognostic scoring system-plus risk distributions were 14% low, 16% intermediate-1, 37% intermediate-2 and 33% high-risk. Driver mutation distributions were 66% *JAK2,* 16%*CALR* type 1/ type1-like, 3%*CALR* type 2/type 2-like, 5% *MPL* and 10% triple-negative. Cytogenetic studies were available in 685 patients and included normal karyotype in 61%. Mutations concomitantly analyzed included *ASXL1* (*n*=467), *SF3B1* (*n*=401), *U2AF1* (*n*=445), *SRSF2* (*n*=461), *TET2* (*n*=179), *EZH2* (*n*=363), *ZRSR2* (*n*=179), *IDH1* (*n*=185) and *IDH2* (*n*=186); frequencies were 38%, 9%, 16%, 15%, 18%, 4%, 11%, 4% and 8%, respectively.

We identified 40 patients (6%) as having been diagnosed with NHS-EMH within a median time of 4 years (range: 0–28 years) from initial diagnosis of PMF. The study population was subsequently stratified according to the presence or absence of NHS-EMH (Table 1). The most common sites involved by NHS-EMH were as follows: lungs (34 cases, 85%), paravertebral region (one case, 2%), perirenal and retroperitoneal region (two cases, 5%), cervical and axillar lymph nodes (two cases, 5%) and pericardium (one case, 2%). The majority of the affected patients (35 cases, 87%) were symptomatic, ascribing complaints correlated to the organs involved by NHS-EMH; the remaining patients (five cases, 13%) were asymptomatic and the diagnosis was incidental. In univariate analysis, NHS-EMH was significantly associated with higher leukocyte count (*P*=0.01; leukocyte count >11 × 10^9^/l; *P*=0.02) and *CALR* mutational status (*P*=0.002). More importantly, none of the above-mentioned other mutations or cytogenetic status showed significant correlation with the occurrence of NHS-EMH in PMF (Table 1). On multivariate analysis, presence of a leukocyte count >11 × 10^9^/l (*P*=0.009) and the presence of *CALR* mutations (*P*=0.0008) retained significance.

The current study highlights for the first time that the presence of molecular and clinical markers, in particular *CALR* mutations and the leukocytosis >11 × 10^9^/l, are associated with NHS-EMH and may possibly contribute to the pathogenesis of PMF-associated NHS-EMH.

Moreover, these findings might allow the identification of those patients who are at risk for the development of NHS-EMH as well as for those who might benefit from an accordingly tailored diagnostic and therapeutic approach.

## Figures and Tables

**Table 1:** Clinical and laboratory features of 704 patients with primary myelofibrosis stratified by presence or absence of NHS-EMH

*Variables*	*All (*n*=704)*	*Presence of NHS-EMH (*n*=40)*	*Absence of NHS-EMH (*n*=664)*	P*-value univariate*	P-*value multivariate*
Age at referall in years median (range)	64 (22–90)	62.5 (38–79)	64 (22–90)	0.1	
Male (%)	454 (64%)	25 (63%)	429 (65%)	0.8	
Hemoglobin, g/dl median (range)	10.5 (5.8–16.7)	10.9 (7.5–13.8)	10.4 (5.8–16.7)	0.4	
Hemoglobin <10 g/dl, *n* (%)	331 (47%)	14 (35%)	317 (48%)	0.1	
Leukocytes, x10^9^/l median (range)	9.0 (0.8–236.1)	12.6 (1.1–105.9)	9 (0.8–236.1)	**0.01**	0.8
Leukocytes >11x10^9^/l, *n* (%)	279 (40%)	23 (58%)	256 (39%)	**0.02**	**0.009**
Leukocytes >25 x10^9^/l, *n* (%)	101 (14%)	7 (18%)	94 (14%)	0.6	
Platelets, x 10^9^/l median (range)	210.0 (10.0–2466.0)	229.5 (13.0–1164.0)	208.5 (10.0–2466.0)	0.9	
Platelets<450x10^9^/l, *n* (%)	595 (85%)	34 (85%)	561 (84%)	0.9	
Platelets <1000 x10^9^/l, *n* (%)	689 (98%)	39 (98%)	650 (98%)	0.9	
Circulating blasts % median (range)	1 (0–15)	0.5 (0–9)	1 (0–15)	0.3	
Presence of constitutional symptoms, *n* (%)	203 (29%)	10 (25%)	193 (29%)	0.6	
Presence of palpable splenomegaly *N* evaluable=680, *n* (%)	509 (75%)	29 (81%)	480 (75%)	0.4	
					
*DIPSS-plus risk N evaluable=685*				0.2	
Low	93 (14%)	6 (15%)	87 (13%)		
Intermediate-1	113 (16%)	10 (25%)	103 (16%)		
Intermediate-2	252 (37%)	16 (40%)	236 (37%)		
High	227 (33%)	8 (20%)	219 (34%)		
					
*Driver mutations*				**0.045**	
* JAK2* mutated, *n* (%)	463 (66%)	20 (50%)	443 (67%)		
* CALR* type 1/type 1-like, *n* (%)	112 (16%)	12 (30%)	100 (15%)		
* CALR* type 2/type 2-like, *n* (%)	22 (3%)	3 (8%)	19 (3%)		
* MPL* mutated, *n* (%)	38 (5%)	2 (5%)	36 (5%)		
Triple negative, *n* (%)	69 (10%)	3 (8%)	66 (10%)		
					
*CALR* mutated, *n* (%)	134 (19%)	15 (38%)	119 (18%)		
Mutated versus unmutated				**0.002**	**0.0008**
					
*Cytogenetic categories N evaluable=685*					
Normal					
Normal versus Abnormal	417 (61%)	26 (65%)	391 (61%)	0.6	
Favorable					
Favorable versus unfavorable*	605 (88%)	38 (95%)	567 (88%)	0.2	
					
*ASXL1,* *N* evaluable=467	178 (38%)	14 (39%)	164 (38%)	0.9	
*SF3B1*, *N* evaluable=401	35 (9%)	4 (13%)	31 (8%)	0.4	
*U2AF1*, *N* evaluable=445	71 (16%)	2 (6%)	69 (17%)	0.1	
*SRSF2*, *N* evaluable=461	70 (15%)	7 (19%)	63 (15%)	0.5	
*TET2*, *N* evaluable=179	32 (18%)	3 (21%)	29 (18%)	0.7	
*EZH2*, *N* evaluable=363	16 (4%)	2 (7%)	14 (4%)	0.5	
*ZRSR2*, *N* evaluable=179	19 (11%)	2 (14%)	17 (10%)	0.6	
*IDH1*, *N* evaluable=185	8 (4%)	0 (0%)	8 (5%)	0.4	
*IDH2*, *N* evaluable=186	14 (8%)	2 (13%)	12 (7%)	0.4	

Abbreviations: DIPSS, dynamic international prognostic scoring system; NHS-EMH, non-hepatosplenic extramedullary hematopoiesis.

Favorable karyotype: normal karyotype or sole or two abnormalities that do not include the above-listed unfavorable cytogenetic abnormalities. Unfavorable karyotype: complex karyotype or sole or two abnormalities that include +8, 7/7q-, i(17q), 5/5q-, 12p-, inv(3) or 11q23 rearrangement. Bold values indicate statistical significant *P* values. * indicates the unfavorable cytogenetic abnormalities listed below in the table.
